# Interaction Effects of Behavioral Inhibition System/Behavioral Activation System and Cost/Probability Biases on Social Anxiety

**DOI:** 10.3389/fpsyg.2019.02536

**Published:** 2019-11-15

**Authors:** Risa Ito, Natsuki Kobayashi, Satoshi Yokoyama, Haruna Irino, Yui Takebayashi, Shin-ichi Suzuki

**Affiliations:** ^1^Graduate School of Human Sciences, Waseda University, Saitama, Japan; ^2^Center for Research on Counseling and Support Services, The University of Tokyo, Tokyo, Japan; ^3^School of Human Sciences, Waseda University, Saitama, Japan; ^4^Graduate School of Biomedical and Health Sciences, Hiroshima University, Hiroshima, Japan; ^5^Akasaka Clinic, Warakukai Medical Corporation, Tokyo, Japan; ^6^Department of Disaster Psychiatry, School of Medicine, Fukushima Medical University, Fukushima, Japan; ^7^Faculty of Human Sciences, Waseda University, Saitama, Japan

**Keywords:** social anxiety, cost bias, probability biases, behavioral inhibition system, behavioral activation system

## Abstract

**Introduction:**

Social anxiety disorder (SAD) symptoms are maintained by cognitive biases, which are overestimations of the severity and likelihood of negative social events (cost/probability biases), and by sensitivity to rewards and punishments that are determined according to behavioral inhibition/behavioral activation systems (BIS/BAS). Cost/probability biases might activate the behavioral immune system and exacerbate the avoidance of social events. Earlier studies have proposed that low BIS or high BAS decrease SAD symptoms; BIS/BAS may even change the effects of cognitive biases on SAD symptoms. Hence, the current study investigates the interaction effects of BIS/BAS and cost/probability biases on SAD symptoms.

**Method:**

Seventy-six Japanese undergraduate students completed the Japanese version of the Liebowitz Social Anxiety Scale (LSAS), which comprises Fear and Avoidance subscales, the BIS/BAS Scale, and the Social Cost Probability Scale.

**Results:**

A multiple regression analysis was performed to examine whether cost/probability biases, BIS/BAS, and their interactions affected SAD symptoms; following this, the main effects of cost bias and BIS were determined for LSAS-Fear (β = 0.64, *p* < 0.001; β = 0.33, *p* < 0.01) and LSAS-Avoidance (β = 0.49, *p* < 0.001; β = 0.35, *p* < 0.01). The interaction effect between cost bias and BAS was significant for LSAS-Avoidance (β = −0.32, *p* < 0.05). Simple slope analysis showed that the slope of cost bias was significant for low-BAS individuals (β = 0.77, *p* < 0.001) but not for high-BAS individuals (β = −0.21, *n.s.*). The interaction effect between probability bias and BAS was significant for LSAS-Avoidance (β = 0.40, *p* < 0.01) as well. Further, simple slope analysis revealed that the slope of probability bias was significant for low-BAS individuals (β = −0.53, *p* < 0.05) but not for high-BAS individuals (β = 0.17, *n.s.*).

**Discussion:**

The study found interesting results with respect to the avoidance of social events. Low-BAS individuals with high cost or low probability biases regarding social events may have a tendency to avoid social events. In contrast, if high-BAS individuals overestimate the cost of social events or underestimate the probability of social events, their anticipation of rewards might prevent them from avoiding social events.

## Introduction

Social anxiety disorder (SAD) is characterized by an excessive fear and avoidance of social situations where individuals feel scrutinized and fearful of being negatively evaluated by others (American Psychiatric Association [APA], 2013). The 12-month prevalence rate of SAD in the United States is approximately 7%, and SAD is associated with an increase in school dropout rates and a decrease in individual well-being, employment, workplace productivity, socioeconomic status, and quality of life (American Psychiatric Association [APA], 2013). Although individuals with SAD experience considerable distress and social impairment, only about half of them in Western societies ever seek treatment, and they tend to do so only after 15–20 years of experiencing the associated symptoms (American Psychiatric Association [APA], 2013). Therefore, it is a public health imperative to understand and reveal the development and maintenance of SAD symptoms.

Cognitive bias is believed to play an important role in the maintenance of SAD symptoms (e.g., Clark and Wells, 1995). Accordingly, Hofmann (2007) reviewed relevant recent laboratory findings and clinical trial results and presented a comprehensive and disorder-specific cognitive behavioral model for SAD. According to this model, individuals with SAD overestimate the severity (cost bias) and likelihood (probability bias) of a negative outcome of a social situation. Thus, individuals with SAD tend to believe that they are in danger of behaving in an inept and unacceptable fashion and that this will result in disastrous consequences. Consequently, they anticipate social mishaps and engage in avoidance and/or safety behaviors (Wells et al., 1995), which reduce unpleasant feelings and prevent future negative outcomes. In addition, these behaviors prevent the disconfirmation of core dysfunctional beliefs (Salkovskis, 1991). In other words, cost/probability biases might activate the behavioral immune system, a motivational system that detects infectious pathogens, triggers disease-relevant emotional and cognitive responses, and promotes avoidance of the infectious pathogens (Schaller and Park, 2011). Further, a recent study reported that the behavioral immune system affects social cognition and social behavior in human societies (Murray and Schaller, 2016). Cost/probability biases might activate individuals’ avoidance of social events, which, in turn, might temporarily relieve their anxiety. This cycle helps maintain SAD symptoms.

However, to date, the ways in which the above-mentioned factors (e.g., cognitive bias, avoidance) interact with one another to develop and maintain SAD symptoms remain unclear. Kimbrel (2008) proposed that the revised Reinforcement Sensitivity Theory (rRST; Gray and McNaughton, 2000) can be used to integrate a wide range of factors into a unified and theoretically driven model of social anxiety. The rRST is a biology-based theory of personality that postulates that three major subsystems of the brain underlie many individual differences in personality, psychopathology, and reinforcement sensitivity. These brain systems are referred to as the Fight–Flight–Freeze System (FFFS), Behavioral Inhibition System (BIS), and Behavioral Approach System (BAS). The FFFS is proposed to motivate avoidance and escape behaviors in response to conditioned and unconditioned aversive stimuli, and BIS is believed to cause anxiety and neuroticism and inhibit behavior by attending to threatening stimuli or the expectation of a threat. BIS is the basis of cognitive biases such as negative beliefs and negative expectations regarding a threatening situation (Kimbrel, 2008). In contrast, BAS is proposed to trigger reward-seeking behavior and impulsivity (Gray, 1970) in individuals, since BAS reflects the factors promoting goal-oriented behavior. Kimbrel (2008) proposed that low BAS represents an additional risk factor for social anxiety. Further, FFFS has been proposed as being useful for animals but less common in human daily life, and FFFS is not important in human research (Kunisato et al., 2007). Therefore, if FFFS is expressed as a human temperament, it is expressed as a system similar to BIS (Pickering et al., 1999). Consistent with the position of contemporary research in this area (e.g., Gray and McNaughton, 2000), Kimbrel (2008) and Kimbrel et al. (2012) took the position that the sensitivity of both the BIS and FFFS were combined. In Kimbrel et al. (2012), the term “BIS–FFFS” is used throughout the paper to refer to self-report measures of BIS based on earlier versions of the theory. Therefore, the current study examines BIS and BAS.

Kimbrel et al. (2012) tested the hypothesis that cognitive biases for negative and threatening social information (memory bias, expectancy bias, belief bias, and perception of threat) mediate the effects of BIS and BAS sensitivity on social anxiety among college students. They found that, under the mediation of these cognitive biases, higher BIS or lower BAS have significant indirect effects on social anxiety. However, the magnitude of the standard partial regression coefficient of BAS on cognitive biases (β = −0.20, *p* < 0.001) was lower than that of BIS on cognitive biases (β = 0.71, *p* < 0.001). In addition, the correlations between BAS and cognitive biases were weak (memory bias; *r* = −0.08, *n.s.*, expectancy bias; *r* = −0.28, *p* < 0.001, belief bias; *r* = −0.23, *p* < 0.001, and perception of threat; *r* = −0.15, *p* < 0.01). Furthermore, Takahashi et al. (2007) reported that BIS and BAS functioned independently of each other. According to these reports, there are not only people who have high-BAS and low-cost/probability bias but also those have high-BAS and high-cost/probability bias. High-BIS and/or low-BAS individuals may overestimate potential social costs and exaggerate the probability of negative outcomes of social events. Further, cost/probability biases might activate the behavioral immune system and exacerbate the avoidance of social events, which may increase the level of SAD symptoms. In contrast, high-BAS individuals may anticipate rewards and prevent themselves from avoiding social events even when they overestimate the social cost and probability of such events; further, this tendency may not increase the level of SAD symptoms. For example, even if a high-BAS individual overestimates the social cost and probability of one’s research presentation at an academic conference, he or she will not avoid but will instead conduct the presentation for growth opportunities and academic achievement. However, to date, no study has directly examined the relationships between BIS/BAS, the cost/probability bias that is strongly related to SAD symptoms, and social anxiety.

Hence, the current study investigates the interaction effects of the BIS/BAS and cost/probability biases on SAD symptoms. The results are expected to contribute to the development of SAD therapies tailored to individual characteristics. Accordingly, we conducted a cross-sectional study to assess BIS/BAS, cost/probability bias, and social anxiety and hypothesized that BIS was positively correlated with cost/probability bias and SAD symptoms. Further, we hypothesized that the coefficient of the interaction effects between BAS and cost bias and that between BAS and probability bias on SAD symptoms are negatively significant. Specifically, for low-BAS individuals, the higher cost/probability bias, the higher the SAD symptoms. Contrastingly, for high-BAS individuals, SAD symptoms do not increase even with an increase in cost/probability bias.

## Materials and Methods

### Participants and Procedures

Participants were 76 undergraduate students (39 women and 35 men, mean age 21.91 ± 5.03 years) of Waseda University, Saitama, Japan. Students were recruited from psychology classes. The inclusion criterion was: (a) being 20 years of age or older. Further, the exclusion criteria were as follows: (a) undergoing counseling, (b) being prescribed medication by a doctor, and (c) having ever continuously visited medical institutions offering psychiatric and psychosomatic medicine. The study did not have invasiveness. Participants were aged over 20 years. Therefore, we omitted the procedures for informed consent in accordance with the recommendations of the Ethical Guidelines for Medical and Health Research Involving Human Subjects. Instead, completion of the questionnaire was considered as informed consent. Further, we provided both verbal and written explanations of informed consent to potential participants based on the recommendations of the Ethical Guidelines for Medical and Health Research Involving Human Subjects. In particular, the questionnaire’s first page contained the following information for participants: the research objective, inclusion and exclusion criteria, consideration of questionnaire completion as the consent to participate, free and voluntary nature of survey participation, information that data will be processed statistically and participant information will be kept anonymous and confidential, and information that participation or non-participation is unrelated to the participants’ class evaluation.

The participants completed the Japanese version of the Liebowitz Social Anxiety Scale (LSAS), which comprises Fear and Avoidance subscales, the BIS/BAS Scale, and the Social Cost Probability Scale (SCOP). The study was approved by the ethics committee of Waseda University.

### Measures

#### Assessment of Social Anxiety Symptoms

The LSAS, comprising 24 items rated on a scale of severity from 0 to 3, is a valid and reliable social anxiety measure (Liebowitz, 1987). The LSAS consists of Fear and Avoidance subscales. The Japanese version of the LSAS (LSAS-J), which was developed to assess social anxiety in the Japanese population, is psychometrically robust (Asakura et al., 2002) and was used for social anxiety evaluation in the current study.

The LSAS items are descriptions of various social events. Some sample items include “Calling in public” and “Expressing my opinion at a meeting.” For each Fear subscale item, the participants were required to rate the degree of fear on a four-point scale. Similarly, for each Avoidance subscale item, they were required to rate the degree of avoidance on a four-point scale.

#### Assessment of Cost/Probability Bias

The SCOP is a 12-item scale of perceived cost/probability bias in social events, with response options ranging from 1 to 5. SCOP is a valid and reliable measure of cost/probability biases (Shirotsuki and Nomura, 2009).

The SCOP has items describing different social events and respondents’ cognition of cost bias in these social events. Some sample items are “I think that my opinion will be misunderstood if I express it in public” and “I think that I will be rejected when I talk with my friend.” To assess cost biases, participants were required to score the following question on a five-point scale: “To what extent does the following idea applies to each situation?” Further, to assess probability biases, the participants had to score the following question on a five-point scale: “To what extent do you think the following idea will come true in each situation?”

#### Assessment of BIS/BAS

Research has proved the validity and reliability of the BIS/BAS Scale, which comprises 20 items rated on a scale of severity from 1 to 4 (Carver and White, 1994). The Japanese version of this scale, which was developed for the Japanese population, is psychometrically robust (Takahashi et al., 2007) and was used to evaluate BIS/BAS in the current study.

### Statistical Analyses

Pearson’s correlation coefficients were calculated to examine the associations between all the variables. After conducting bivariate analysis, we conducted a multiple regression analysis to examine whether cost/probability biases, BIS/BAS, and their interactions affected SAD symptoms. We used SPSS version 24.0 to analyze the data.

## Results

### Descriptive Statistics

Table 1 presents the characteristics of the 76 participants. The numbers of participants rated for each severity level of SAD symptoms were as follows: less than mild (0–43 points; *n* = 31), mild (44–79 points; *n* = 37), moderate (80–101 points; *n* = 8), and severe (over 102 points; *n* = 0). Eighty-nine percent of the participants were rated as having mild or less than mild symptoms. In addition, the means and standard deviations of each of the descriptive variables were similar to those of earlier findings on healthy Japanese people (Takahashi et al., 2007; Shirotsuki et al., 2010).

**TABLE 1:** Demographic data for participants and the results of normality test (*N* = 76).

	***Mean***	***SD***	**Kolmogorov–Smirnov test**	
			
			***Z***	***p*-Value**
**Social anxiety**				
LSAS-Total	50.46	23.13	0.08	0.76
LSAS-Fear	27.97	12.26	0.08	0.67
LSAS-Avoidance	22.49	12.06	0.06	0.93
**Cognitive bias**				
Cost bias	31.25	8.41	0.09	0.60
Probability bias	31.49	9.00	0.07	0.88
**Temperament**				
BIS	20.70	4.00	0.08	0.73
BAS	37.67	6.76	0.07	0.83

*LSAS = liebowitz social anxiety scale, BIS = behavioral inhibition system, BAS = behavioral activation system.*

We performed the Kolmogorov–Smirnov test to examine the normality of variables (Table 1). The results revealed that all the data were not significant. Therefore, it found that all the variables were normally distributed.

### Correlation Between Variables

Pearson’s correlation analysis was applied to examine the associations among all the variables (Table 2). The results revealed significant positive correlations between fear symptoms of social anxiety and cost bias (*r* = 0.61, *p* < 0.001), probability bias (*r* = 0.27, *p* = 0.02), and BIS (*r* = 0.41, *p* < 0.001). In addition, there were significant positive correlations between avoidance symptoms of social anxiety and cost bias (*r* = 0.42, *p* < 0.001), probability bias (*r* = 0.29, *p* = 0.01), and BIS (*r* = 0.44, *p* < 0.001). Moreover, there were significant positive correlations between BIS and cost bias (*r* = 0.37, *p* = 0.001), between BIS and probability bias (*r* = 0.42, *p* < 0.001), and between BIS and BAS (*r* = 0.32, *p* = 0.004).

**TABLE 2:** Pearson’s correlations between variables (*N* = 76).

	**1**	**2**	**3**	**4**	**5**	**6**	**7**
**Social anxiety**							
1 LSAS-Total	−	0.95^∗∗∗^	0.95^∗∗∗^	0.54^∗∗∗^	0.29^∗^	0.44^∗∗∗^	−0.01
		[90.98]^∗^	[90.98]^∗^	[22.76]^∗^	[00.54]^∗^	[09.70]^∗^	
2 LSAS-Fear		−	0.81^∗∗∗^	0.61^∗∗∗^	0.27^∗^	0.41^∗∗∗^	−0.04
			[63.91]^∗^	[31.80]^∗^	[05.47]^∗^	[05.67]^∗^	
3 LSAS-Avoidance			–	0.42^∗∗∗^	0.29^∗^	0.44^∗∗∗^	0.01
				[07.68]^∗^	[07.48]^∗^	[08.69]^∗^	
**Cognitive bias**							
4 Cost bias				–	0.52^∗∗∗^	0.37^∗∗^	0.003
					[19.75]^∗^	[09.60]^∗^	
5 Probability bias						0.42^∗∗∗^	0.04
						[07.68]^∗^	
**Temperament**							
6 BIS						–	0.32^∗∗^
							[03.56]^∗^
7 BAS							–

*LSAS = liebowitz social anxiety scale, BIS = behavioral inhibition system, BAS = behavioral activation system. Values enclosed in parentheses represent the 95% confidence interval for each parameter. ^∗^*p* < 0.05, ^∗∗^*p* < 0.01, ^∗∗∗^*p* < 0.001.*

### Testing Assumptions of Multiple Regression

**FIGURE 1:**
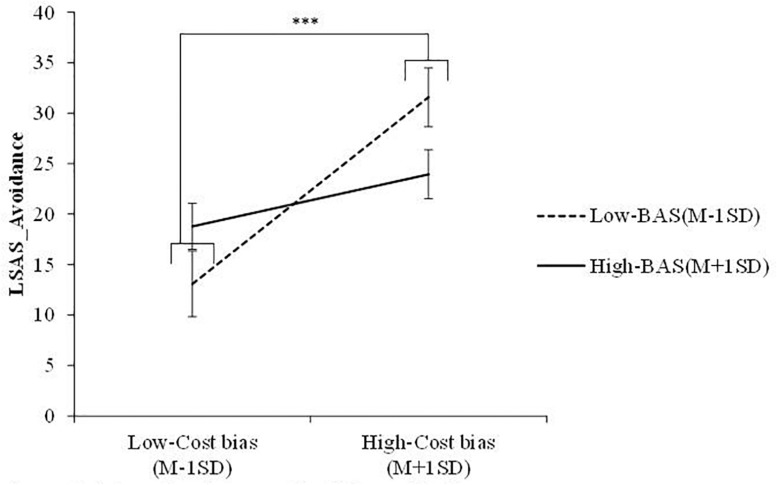
Interaction between cost bias and BAS. *LSAS* = liebowitz social anxiety scale; *BAS* = behavioral activation system. ^∗∗∗^*p* < 0.001.

**FIGURE 2:**
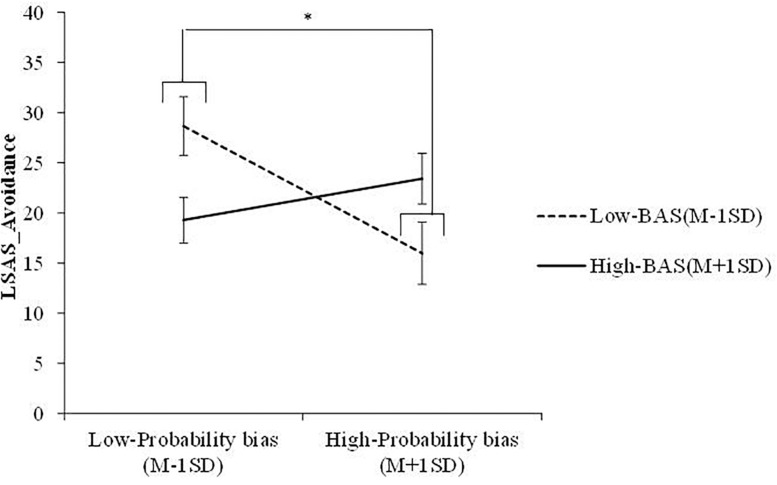
Interaction between probability bias and BAS. *LSAS* = liebowitz social anxiety scale; *BAS* = behavioral activation system. ^∗^*p* < 0.05.

A multiple regression analysis was conducted to examine whether cost/probability biases, BIS/BAS, and the interactions between these variables predicted SAD symptoms (Table 3). Accordingly, significant main effects of cost bias and BIS were found for LSAS-Fear (β = 0.64, *p* < 0.001; β = 0.33, *p* < 0.01) and LSAS-Avoidance (β = 0.49, *p* < 0.001; β = 0.35, *p* < 0.01). The results suggested that an increase in cost bias and BIS significantly increased SAD symptoms. Further, the interaction effect between cost/probability bias and BAS was not significant for LSAS-Fear (cost bias: β = −0.05, *n.s.*; probability bias: β = 0.18, *n.s.*), whereas that between cost bias and BAS was significant for LSAS-Avoidance (β = −0.32, *p* < 0.05). Simple slope analysis showed that the slope of cost bias was significant for low-BAS (β = 0.77, *p* < 0.001) but not high-BAS (β = 0.21, *n.s.*) individuals. Further, the results revealed that the combination of low BAS and high cost bias increased avoidance. The interaction effect between probability bias and BAS was significant for LSAS-Avoidance (β = 0.40, *p* < 0.01), as well. Further, simple slope analysis showed that the slope of probability bias was significant for low-BAS (β = −0.53, *p* < 0.05) but not high-BAS (β = 0.17, *n.s.*) individuals. The results showed that the combination of low BAS and low probability bias increased avoidance. Figures 1, 2 illustrate these results.

**TABLE 3:** The results of multiple regression analysis (*N* = 76).

**Variables**	**LSAS-Fear**		**LSAS-Avoidance**		**VIF**
			
	**β**	**95% Confidence interval**	****β****	**95% Confidence interval**	
**Cognitive bias**					
Cost bias	0.64^∗∗∗^	0.40–0.88	0.49^∗∗∗^	0.23–0.75	1.87
Probability bias	−0.24^†^	−0.48–0.00	–0.18	−0.44–0.08	1.85
**Temperament**					
BIS	0.33^∗∗^	0.11–0.55	0.35^∗∗^	0.11–0.58	1.51
BAS	–0.10	−0.29–0.10	–0.04	−0.25–0.17	1.20
**Cognitive bias × temperament**					
Cost bias × BAS	–0.05	−0.29–0.19	−0.32^∗^	−0.58–−0.06	1.84
Probability bias × BAS	0.18	−0.06–0.42	0.40^∗∗^	0.14–0.66	1.84
Cost bias × BIS	0.17	−0.04–0.38	0.12	−0.11–0.34	1.43
Probability bias × BIS	–0.15	−0.36–0.06	0.01	−0.22–0.24	1.45
*R*^2^	0.42^∗∗∗^		0.31^∗∗∗^		

*LSAS = liebowitz social anxiety scale, BIS = behavioral inhibition system, BAS = behavioral activation system, VIF = variance inflation factor. ^†^*p* < 0.10, ^∗^*p* < 0.05, ^∗∗^*p* < 0.01, ^∗∗∗^*p* < 0.001.*

## Discussion

The current study examined the interaction effects between BIS/BAS and cost/probability biases on SAD symptoms. The results of Pearson’s correlation analyses revealed significant positive correlations between BIS and cost bias, probability bias, and SAD symptoms. Further, multiple regression analysis detected significant interaction effects between cost bias and BAS and between probability bias and BAS for avoidance of social events. Simple slope analysis revealed that the slope of cost/probability bias was significant for low-BAS but not high-BAS individuals. In particular, low-BAS individuals who estimate that there will be a high cost or low probability of social events tend to avoid them. On the other hand, even if high-BAS individuals overestimate the cost or probability of social events, the anticipation of positive outcome occurrence may supersede their tendency to avoid social events. These results were consistent with the first hypothesis that BIS is positively correlated with cost/probability bias and SAD symptoms. Further, the second hypothesis was that low-BAS individuals with high-cost/probability bias presented increased SAD symptoms. The results of the multiple regression analysis of avoidance of social events partially confirmed the second hypothesis, whereas high-BAS individuals did not experience increased SAD symptoms regardless of the degree of their cost/probability bias.

Regarding the first hypothesis that BIS is positively correlated with cost/probability bias and SAD symptoms, the current results conform to the findings obtained by other researchers. BIS is proposed to trigger response to threatening stimuli or expectation of such stimuli. Thus, high BIS may cause cognitive biases such as negative belief and negative expectation in threatening situations (Kimbrel, 2008) and high-BIS individuals might tend to avoid danger, in general (Peters and Slovic, 2000). Avoidance of danger might mediate the relationship between BIS and SAD symptoms (Lorian and Grisham, 2010). Therefore, high-BIS individuals would overestimate the potential social costs and exaggerate the probability of negative outcomes of social events. The cost/probability biases might activate the behavioral immune system and exacerbate the individuals’ avoidance of social events. Avoidance of danger might strengthen the individuals’ cognition of negative outcomes (Maner and Schmidt, 2006), and SAD symptoms might be negatively reinforced and maintained in these individuals.

The second hypothesis, that the coefficient of the interaction effects between BAS and cost bias and between BAS and probability bias on SAD symptoms is negatively significant, was confirmed only for cost bias on the avoidance symptoms of SAD. In addition, regarding probability bias, the results showed that a low probability bias might increase the avoidance symptoms of SAD; this result was inconsistent with our hypothesis and earlier reports (e.g., Shirotsuki et al., 2010).

First, we consider the results on cost bias. BAS has been proposed to promote goal-oriented behavior (Gray, 1970). Based on an earlier study on how a low BAS represented an additional risk factor for social anxiety (Kimbrel, 2008), if low-BAS individuals overestimate the cost of social events, their cost bias might activate the behavioral immune system and exacerbate avoidance of social events. Avoidance of danger might strengthen cognitions of negative outcomes (Maner and Schmidt, 2006) and help maintain SAD symptoms. On the other hand, high-BAS individuals might react strongly to a reward and attempt to achieve it. Even if high-BAS individuals overestimate the cost of social events and fear social events, they might try to attain the reward by experiencing the social events. Consequently, the attempt would indirectly decrease avoidance of social events.

Second, the relationship between probability bias and avoidance of social events was inconsistent with the findings of earlier studies, according to which overestimating the likelihood of a social situation’s negative outcome would increase SAD symptoms (Hofmann, 2007). Since this result pertains to a rare occurrence, there is a possible that multicollinearity occurred. In order to confirm whether multicollinearity is an issue, Variance Inflation Factor (VIF) was calculated. The results showed that all values of VIF were less than 2, so multicollinearity is likely not an issue. Nevertheless, in order to confirm the robustness of the results, further studies should recruit a larger sample and examine the current study’s findings again. It has been proposed that probability bias leads to social anxiety through factors other than those directly affecting social anxiety (Shirotsuki and Nomura, 2009). Shirotsuki et al. (2010) showed that probability bias increased cost bias and indirectly affected avoidance and anxiety. However, according to the correlation between cost bias and probability bias and the results of the interaction effect between cost bias and BAS on avoidance, there is a possibility that probability bias also leads to avoidance through other factors. Research on cognitive strategies suggests that individuals with unjustified optimism who have a negative cognition of past performance but have set positive expectations for the future tend to adopt self-handicapping or avoidance coping styles (Mitsunami, 2010). In other words, there are cases where individuals estimate future risk low and therefore are less motivated to manage current problems. Reducing future risk may be similar to situations with low probability bias. Our results here suggest that low-BAS individuals with low probability bias may engage in cognitive strategies that downplay future risk, believing that terrible outcomes are rare. For example, students might think that even if they are absent from a public presentation, they are unlikely to fail the class. Because of this bias, they may have little motivation to be exposed to stressful social situations. In contrast, low-BAS individuals with high probability bias may believe the future is very risky. For example, students might think that the absence from a presentation is highly likely to result in failing the class. This mentality drives social engagement as a way to prevent negative outcomes. Therefore, future studies should examine in more detail whether cognitive strategies reducing future risk could influence social avoidance.

Regarding why a difference was observed for avoidance but not fear, Shirotsuki et al. (2010) clarified that avoidance of social events increases fear of social events. Further, McManus et al. (2008) proposed that safety behaviors in SAD individuals might lead to higher anxiety. In contrast, Moitra et al. (2008) showed that anxiety about social events exacerbates avoidance and finally results in depression. However, in the current study, the main outcome was SAD symptoms rather than depression. Therefore, we have adopted the former model, according to which avoidance of social events increases the fear of social events. Since fear of social events might be a secondary reaction following avoidance of events, no difference can be observed in fear of social events in a cross-sectional study.

In addition, the results of Pearson’s correlation analyses revealed significant positive correlations between BIS and BAS. Earlier studies have revealed that BIS and BAS are independent of each other (Carver and White, 1994). On the other hand, some studies have also reported correlations between BIS and BAS (e.g., Takahashi et al., 2007), as well. Takahashi et al. (2007) reported a weak correlation (*r* = 0.12, *p* < 0.05) between the BIS and BAS. This weak correlation might be caused by the effect of spurious correlation. Because BIS and BAS were assumed to correlate positively with neuroticism, the partial correlation coefficient between BIS and BAS controlling neuroticism was calculated. Results showed that there was no significant partial correlation coefficient (*pr* = −0.05, *p* > 0.50). Since the current study used the scale developed by Takahashi et al. (2007), the correlation between BIS and BAS in this study might also be affected by the effect of spurious correlation. Future studies should further assess neuroticism and examine the current study’s findings.

### Limitations and Future Directions

The current study has three main limitations. The first is our use of a student sample. We believe that future studies may benefit from replicating the findings using a clinical sample. Second, we did not conduct a social threat manipulation. Kimbrel (2008) proposed that socially threatening cognitions might occur only among socially anxious individuals under conditions of imminent social threat. Future studies should add a social threat manipulation and contextually examine the current study’s findings. Third, the current study used a relatively small sample size. In the multiple regression analysis, although there were eight predictor variables in the model, the number of participants was only 79 (less than 10 participants per predictor), which is generally assumed to be the absolute minimum number (e.g., Wilson Van Voorhis and Morgan, 2007). Hence, further studies should recruit a larger sample and examine the current study’s findings.

## Conclusion

This study found interesting results regarding avoidance of social events. If low-BAS individuals overestimate the cost or underestimate the probability of social events, they tend to avoid them. On the other hand, in the case of overestimation or underestimation by high-BAS individuals, their desire to receive rewards tends to supersede their tendency to avoid social events. Although this topic requires additional research, our findings imply that the cost/probability bias toward social events may be the mechanism through which BIS and BAS exert their influence on SAD symptoms. Future studies should examine the change in avoidance symptoms of SAD while addressing cost/probability bias.

## Data Availability Statement

The datasets generated for this study are available on request to the corresponding author.

## Ethics Statement

This study was carried out in accordance with the recommendations of the Ethical Guidelines for Medical and Health Research Involving Human Subjects, Ministry of Education, Culture, Sports, Science and Technology and Ministry of Health, Labor and Welfare. The current study did not involve invasiveness. All the participants were over 20 years of age. Therefore, we omitted the procedures concerning informed consent in accordance with the recommendations of the Ethical Guidelines for Medical and Health Research Involving Human Subjects. Further, completion of the questionnaire was considered informed consent. We provided both verbal and written explanations on informed consent to potential participants based on the recommendations of the Ethical Guidelines for Medical and Health Research Involving Human Subjects. Further, the participants were informed about the study’s objectives, notified of the voluntary nature of participation, and assured of their data’s anonymity and confidentiality. The protocol was approved by the Research Ethics Committee of Waseda University.

## Author Contributions

RI, NK, SY, and SS developed the research idea. RI, NK, SY, HI, and YT performed the data collection. RI performed the data analyses and wrote the first draft. All authors contributed to the revision of the manuscript.

## Conflict of Interest

The authors declare that the research was conducted in the absence of any commercial or financial relationships that could be construed as a potential conflict of interest.
